# Correction: A novel GTP-binding protein–adaptor protein complex responsible for export of Vangl2 from the *trans* Golgi network

**DOI:** 10.7554/eLife.01328

**Published:** 2013-08-28

**Authors:** Yusong Guo, Giulia Zanetti, Randy Schekman

Guo Y, Zanetti G, Schekman R. 2013. A novel GTP-binding protein–adaptor protein complex responsible for export of Vangl2 from the *trans* Golgi network. *eLife* **2**:e00160. doi: http://dx.doi.org/10.7554/eLife.00160. Published 08 January 2013

An error was identified in Figure 2. The fourth panel of the original Figure 2C was incorrectly identified as having been probed with an antibody against µ1 subunit to AP-1, but was instead mistakenly reproduced in a second, inverted exposure of the panel above.

The filter transfer of a protein gel was processed into three strips, one of which was then probed with an antibody against the γ1 subunit of AP-1 (the third panel of Figure 2C). Another strip was probed with an antibody against the µ1 subunit of AP-1, but the reaction failed and no signal was produced. Because of a mix up of strips upon repeating the incubation with the anti-µ1 antibody, the strip that had been reacted with the anti-γ1 antibody was mistakenly used. The fourth panel shows the subsequent exposure, which reflects the signal from the previous probing with the γ1 subunit antibody, as we had not removed the previous signal. Because of an error with labeling the molecular weight marker, the panel is also inverted relative to the panel above it.

We saved all three strips and now show results from probing the third strip with anti-µ1 antibody. We apologize for the original mistake.

The corrected figure is shown here:

**Figure 1:**
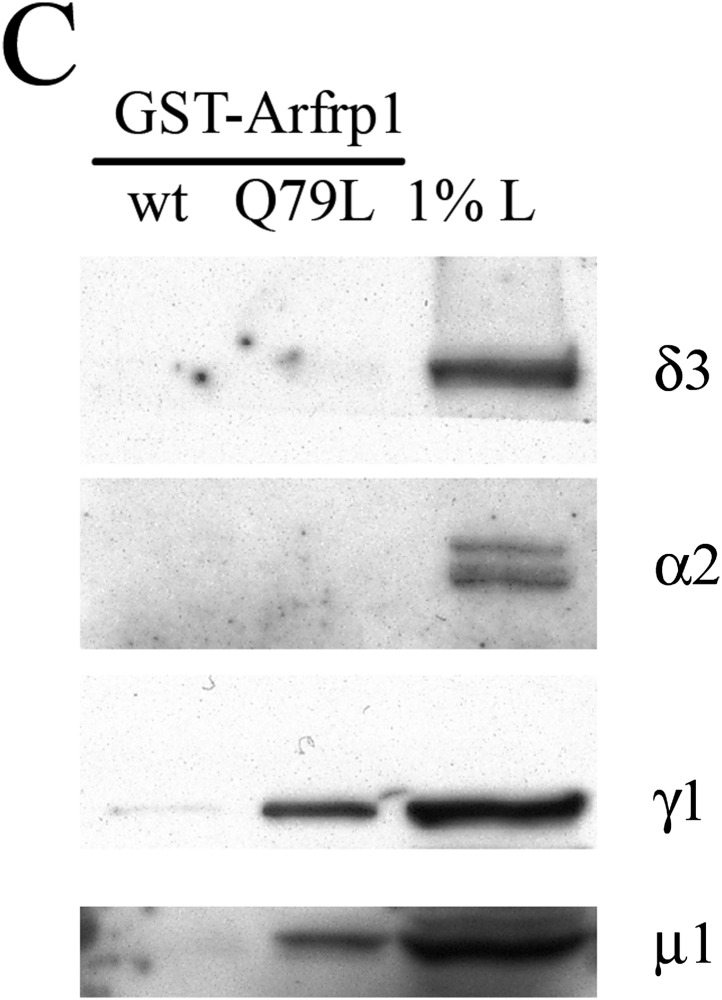


The originally published figure is also shown for reference:

**Figure 2:**
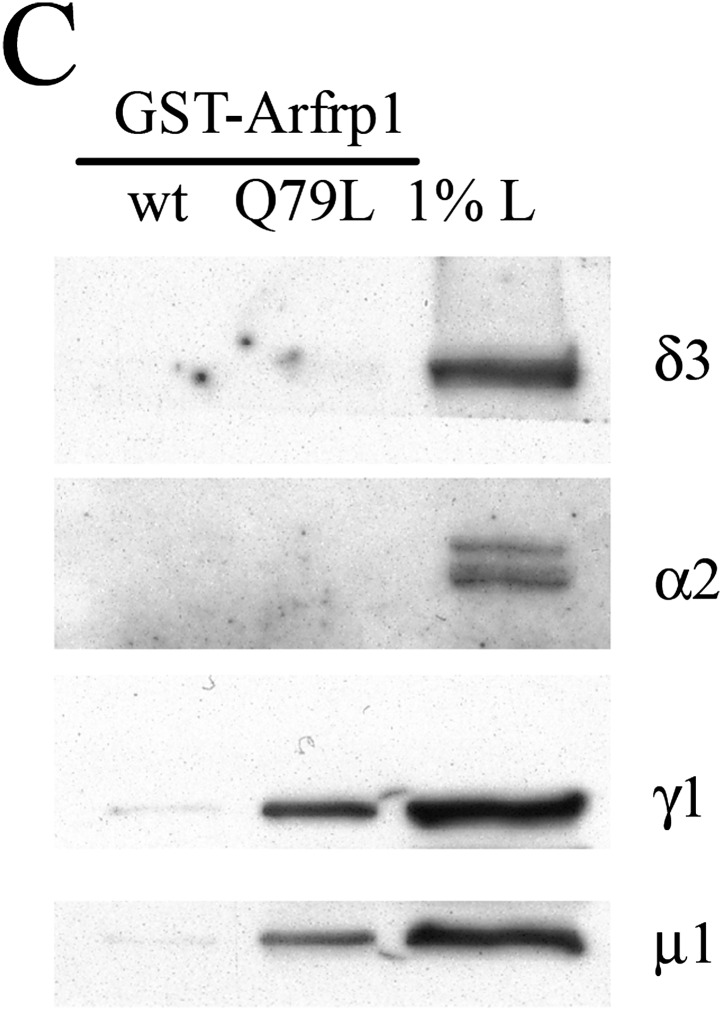


The article has been corrected accordingly.

